# Influence of Pontic Length on the Structural Integrity of Zirconia Fixed Partial Dentures (FPDs)

**DOI:** 10.3390/jfb16040116

**Published:** 2025-03-25

**Authors:** Tareq Hajaj, Ioana Elena Lile, Ioana Veja, Florina Titihazan, Mihai Rominu, Meda Lavinia Negruțiu, Cosmin Sinescu, Andreea Codruta Novac, Serban Talpos Niculescu, Cristian Zaharia

**Affiliations:** 1 Department of Prostheses Technology and Dental Materials, Faculty of Dentistry, Victor Babes University of Medicine and Pharmacy, 2 Eftimie Murgu Sq., 300041 Timisoara, Romania; tareq.hajaj@umft.ro (T.H.); florina.titihazan@umft.ro (F.T.); rominu.mihai@umft.ro (M.R.); negrutiu.meda@umft.ro (M.L.N.); sinescu.cosmin@umft.ro (C.S.); cojocariu.andreea@umft.ro (A.C.N.); cristian.zaharia@umft.ro (C.Z.); 2 Research Center in Dental Medicine Using Conventional and Alternative Technologies, Faculty of Dental Medicine, Victor Babes University of Medicine and Pharmacy of Timisoara, 9 Revolutiei 1989 Ave, 300070 Timisoara, Romania; 3 Department of Dental Medicine, Faculty of Dentistry, “Vasile Goldis” Western University of Arad, Str. Liviu Rebreanu 86, 310045 Arad, Romania; lile.ioana@uvvg.ro; 4 Department of Oral and Maxillofacial Surgery, Faculty of Dentistry, Victor Babes University of Medicine and Pharmacy, 2 Eftimie Murgu Sq., 300041 Timisoara, Romania

**Keywords:** zirconia restorations, pontic length, fracture resistance, dental bridges, mechanical strength, CAD/CAM, prosthodontics

## Abstract

Objective: This study aims to evaluate the influence of pontic length and design on the fracture resistance of zirconia fixed dental prostheses (FDPs). By assessing different span lengths under controlled mechanical loading conditions, the research seeks to provide insights into optimizing the structural integrity of zirconia dental bridges. Materials and Methods: A total of 20 zirconia bridges were fabricated and tested in vitro. Ten bridges were designed to replace a single missing molar (tooth 46), with a pontic span of 11 mm, while the remaining ten were crafted for two missing teeth (35 and 36), featuring a longer pontic span of 17 mm. The zirconia frameworks were milled using the Wieland Zenotec^®^ Select Hybrid system and cemented onto metal abutments with Voco Meron Plus QM resin-reinforced glass ionomer cement. The specimens were subjected to occlusal loading using a ZwickRoell ProLine Z005 testing machine at a crosshead speed of 1 mm/min until fracture occurred. Results: The mechanical testing revealed a significant correlation between pontic length and fracture resistance. The mean fracture resistance for three-unit bridges (single pontic) was 3703 N, whereas four-unit bridges (double pontic) exhibited a significantly lower resistance of 1713 N. These findings indicate that increased span length reduces the fracture resistance of zirconia restorations due to higher stress accumulation and reduced rigidity. Conclusions: This study underscores the importance of pontic length and design in determining the fracture resistance of zirconia restorations. Shorter spans exhibit greater structural stability, reinforcing the need for careful treatment planning when designing multi-unit zirconia bridges. By optimizing bridge parameters, clinicians can improve clinical outcomes and extend the longevity of zirconia prostheses in restorative dentistry.

## 1. Introduction

Zirconia has fundamentally transformed restorative dentistry due to its outstanding biocompatibility, favorable esthetics, and superior mechanical properties. Zirconia is a crystalline ceramic derived from zirconium oxide and is distinguished by remarkable strength, toughness, and fracture resistance. These characteristics make zirconia an ideal material for a wide array of dental applications, including bridges, crowns, and implants [1,2,3,4]. Among these, zirconia bridges have gained recognition as reliable solutions for replacing multiple missing teeth, fulfilling both functional and esthetic requirements essential for patient satisfaction.

As demand increases for restorations that are both long-lasting and natural-looking, understanding the mechanical resilience of zirconia bridges under masticatory forces becomes crucial. During mastication, dental bridges are subjected to complex loading regimes, including dynamic occlusal forces, torsional stresses, and shear forces [5,6,7]. The mechanical performance of zirconia bridges is vital for their longevity and clinical success. Unlike traditional metal-based bridges that provide strength at the expense of esthetics, zirconia bridges offer a unique combination of high strength and lifelike appearance, blending seamlessly with the patient’s dentition and making them a preferred choice in many clinical scenarios [8,9,10,11].

Research has shown that numerous factors influence the mechanical resistance of zirconia bridges. These factors include the bridge design, connector dimensions, the thickness and length of the framework, and the fabrication process used. The span of the bridge (the distance between abutment supports) is particularly significant: longer spans present challenges in rigidity and stress distribution that can affect performance and longevity of the restoration [2,12,13,14]. An increase in span length (for instance, in a fixed partial denture framework of six or more units) has been associated with decreases in marginal and internal fit, potentially compromising the restoration’s effectiveness and longevity [15]. Therefore, optimizing span length and connector geometry is essential to enhance the performance and reliability of zirconia bridges and to reduce the risk of mechanical complications such as framework fracture or debonding.

Advancements in computer-aided design and manufacturing (CAD/CAM) have enabled precise customization of zirconia frameworks. High-precision milling and improved zirconia materials (including high-translucency and multi-layered zirconia) allow clinicians to achieve desired esthetics while maintaining the necessary strength and durability [15]. These developments have improved the fit and function of zirconia restorations in clinical practice. Additionally, interdisciplinary collaboration—dentists working closely with dental technicians—has become important in optimizing design and fabrication processes for complex restorations. By integrating research findings into practice, dental professionals can create highly customized, durable zirconia bridges that meet patient expectations for both esthetic and functional outcomes [5].

The clinical implications of zirconia bridge resistance are incredibly important for dental practitioners, as these factors directly influence treatment planning and material selection [16,17,18,19,20]. A comprehensive understanding of how various factors interact and impact mechanical performance can greatly assist dentists in making well-informed decisions regarding the design and application of zirconia bridges. This knowledge is crucial for ensuring that the restorations not only meet esthetic demands but also withstand the functional stresses encountered in the oral environment.

In addition to the mechanical and esthetic advantages, the evolution of zirconia technology also emphasizes the importance of interdisciplinary collaboration in dental practice [21]. As dental professionals increasingly embrace advancements in materials science, they must also work closely with dental technicians to optimize the design and fabrication processes. This collaboration can lead to the development of highly customized solutions that address the unique needs of each patient. Furthermore, ongoing education and training in the latest techniques and technologies will empower practitioners to make informed decisions, ultimately enhancing the quality of care provided. The integration of research findings into clinical practice can foster innovation, ensuring that zirconia bridges not only meet but exceed patient expectations, paving the way for more effective and durable dental restorations in the future [22].

While zirconia is widely used for FDPs due to its strength and biocompatibility, the specific influence of pontic length on its structural integrity remains insufficiently explored. Studies have shown that longer pontic spans may introduce mechanical challenges such as higher stress concentrations, increased flexural strain, and potential connector failure [8,9]. However, existing literature primarily focuses on connector design and material properties, with limited quantitative analysis of how pontic length alone affects fracture resistance [10,11]. Understanding this relationship is crucial for optimizing zirconia bridge design and ensuring long-term success in clinical applications.

Ultimately, understanding the resistance of zirconia bridges is vital not only for optimizing individual patient outcomes but also for improving the overall quality of dental restorations provided to patients across diverse clinical scenarios. Through continuous research and innovation in the field of restorative dentistry, the dental profession can refine techniques and materials to ensure that zirconia bridges remain a leading choice for both clinicians and patients alike [23]. This ongoing commitment to improving dental materials and methodologies will undoubtedly enhance the standard of care available in restorative dentistry, ensuring patient satisfaction and successful long-term outcomes.

Aim: The primary objective of the current study is to thoroughly investigate the relationship between span length and the physical resistance of zirconia restorations when exposed to occlusal stress. This study aims to evaluate the influence of pontic length on the fracture resistance of zirconia fixed dental prostheses (FDPs), to determine whether an increase in pontic span negatively impacts mechanical strength, potentially leading to higher failure rates in extended zirconia bridges.

Hypothesis: We hypothesize that longer pontic spans will exhibit significantly lower fracture resistance than shorter spans due to increased flexural stress and decreased structural rigidity in zirconia FDPs.

## 2. Materials and Methods

This study was carried out at the Faculty of Dentistry of the “Victor Babes” University of Medicine and Pharmacy of Timisoara, within Department of Prostheses Technology and Dental Materials and also in collaboration with the Department of Materials Resistance of the Faculty of Mechanics of the Polytechnic University of Timisoara.

### 2.1. Sample Preparation

In the first phase of the project, a mandible model was scanned in a dental laboratory. This model had one tooth missing on one side and two teeth missing on the other side. The goal of the scanning was to create a digital version of the model. Specifically, the missing teeth were 35 and 36 on the lower left side, while tooth 46 was missing on the opposite side. This scanning process was important for ensuring an accurate digital copy, which would be useful for further study and dental applications.

The model was scanned using the Smart Optics Vinyl (Sensortechnik GmbH, Meerbusch, Germany) laboratory scanner, which was chosen for its remarkable versatility and optimal imaging quality. The scanner provides ample space for accommodating various dental objects. This includes everything from individual dental segments to complete jaw models, as well as both full and partial impressions, even for toothless jaws.

**Figure 1:**
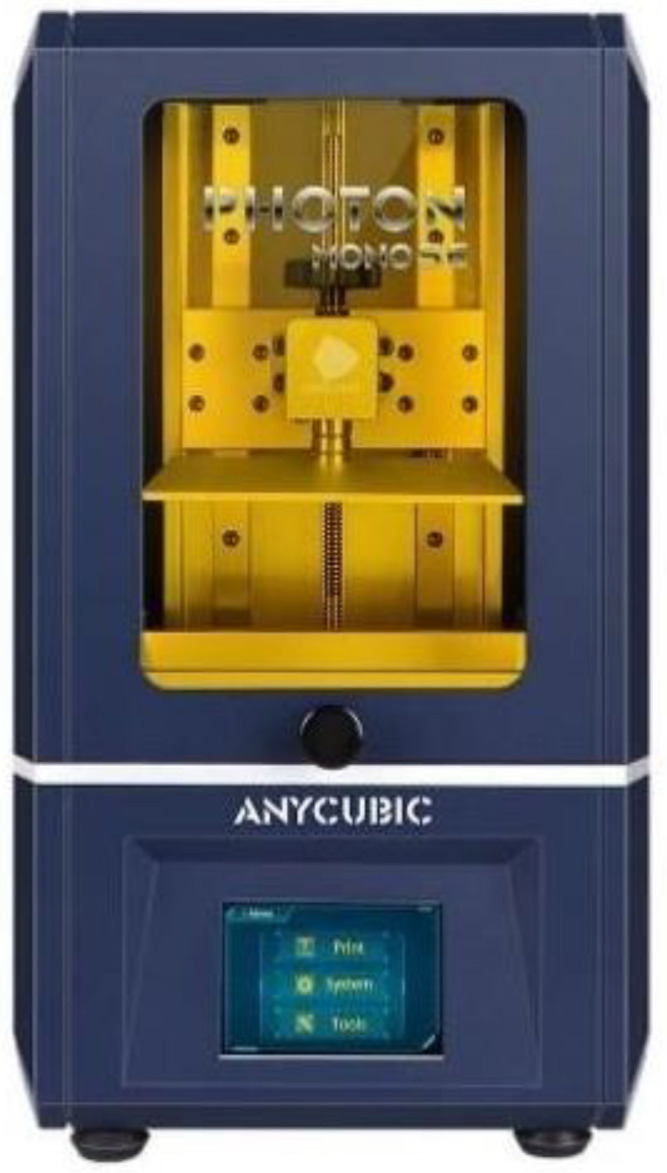
Anycubic Photon Mono SE 3D printer.

**Figure 2:**
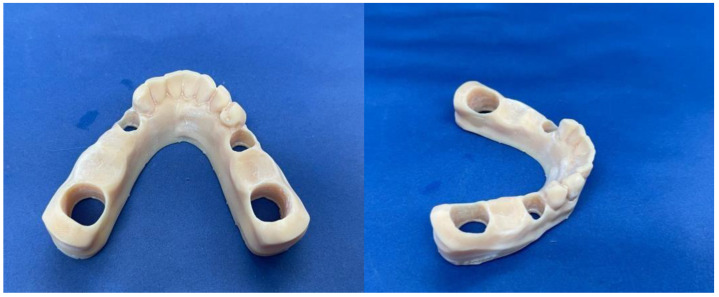
Mandibular 3D printed model.

Afterwards, the 3D printed Anycubic Photon Mono SE (Shenzhen Anycubic Technology Co., Ltd., Shenzhen, China) (Figure 1), was used to print the resin model (Figure 2) used for the study.

**Figure 3:**
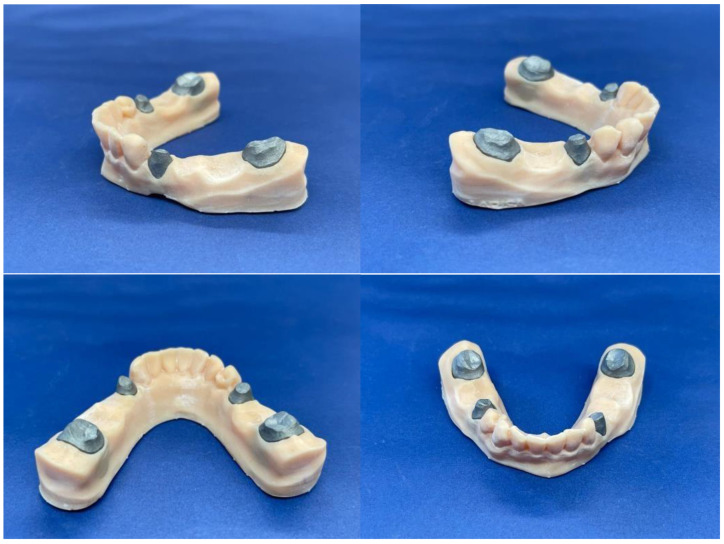
The model with the inserted abutments.

Upon completion of the model, metal removable abutments were fabricated. This material was selected to ensure sufficient durability for all subsequent mechanical loading tests and to mitigate the risk of fracturing the abutment teeth. The metal abutments, designed to support the zirconia frameworks, were prepared to accommodate the prosthetic loading requirements (Figure 3).

The subsequent step in the process involved the manufacturing of the zirconia bridges. The pontics were meticulously crafted utilizing CAD-CAM technology exocad DentalCAD v3.1 (exocad GmbH, Darmstadt, Germany), which allowed for precise control over the design thickness. This design approach was implemented to guarantee both strength and durability across two different scenarios: one missing tooth versus two missing teeth. Every parameter involved in this process adhered strictly to the manufacturer’s recommendations, ensuring optimal performance and reliability in the final product.

**Figure 4:**
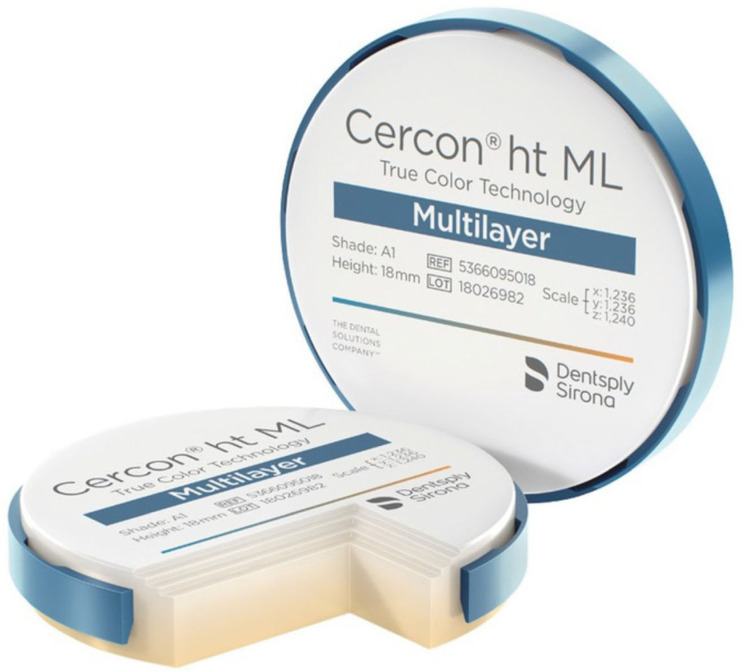
Cercon^®^ ht ML by Dentsply Sirona.

For this purpose, the material selected was Dentsply Sirona’s Cercon^®^ ht ML (Dentsply Sirona, Charlotte, NC, USA), as illustrated in Figure 4. Cercon^®^ ht ML is a sophisticated, multi-layered hybrid zirconia that effectively combines remarkable strength with an esthetically pleasing, natural appearance. This innovative material offers an impressive blend of high bending strength and a lifelike tooth-like appearance, all within a single disk that features a gradient design. Specifically, the material exhibits a bending strength of 1200 MPa in the dentin region, which gradually decreases to 750 MPa at the incisal edge. This unique gradient not only enhances the functional capabilities of the zirconia bridges but also ensures that they closely resemble the natural teeth they are designed to replace, thereby meeting both esthetic and performance demands in restorative dentistry [16].

**Figure 5:**
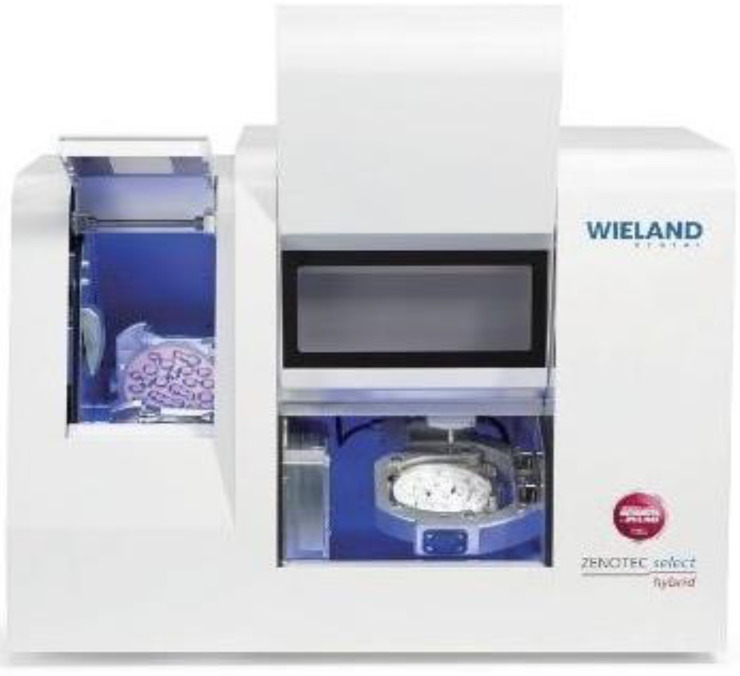
Zenotec^®^ Select Hybrid zirconia milling machine.

A total of twenty CAD-CAM crafted restorations were created specifically for the purpose of mechanical testing, and these were divided into two distinct groups. Each group contained ten specimens that were allocated to correspond with specific bridge lengths. The prosthetic restorations were produced in a specialized laboratory environment, employing advanced technology for the milling of zirconia. This process utilized the Wieland Zenotec^®^ Select Hybrid system (ZwickRoell GmbH & Co. KG, Ulm, Germany), as depicted in Figure 5.

Among these carefully manufactured restorations, ten bridges were specifically designed to replace the missing tooth 46, effectively addressing an edentulous space that measures precisely 11 mm. On the other hand, the remaining ten restorations were skillfully crafted to cater to the third quadrant of the dental arch. These were designed to accommodate the absence of both teeth 35 and 36, resulting in a total pontic length of 17 mm.

**Figure 6:**
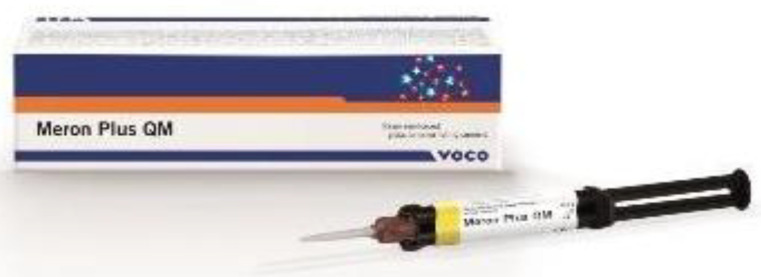
Meron Plus QM^®^.

The next step was to cement the zirconia prosthetic restorations on the model removable abutments using Voco Meron Plus QM (VOCO GmbH, Cuxhaven, Germany) reinforced resin-reinforced glasionomer cement (Figure 6). The cementing protocol was strictly performed using the producer’s recommendations [17].

**Figure 7:**
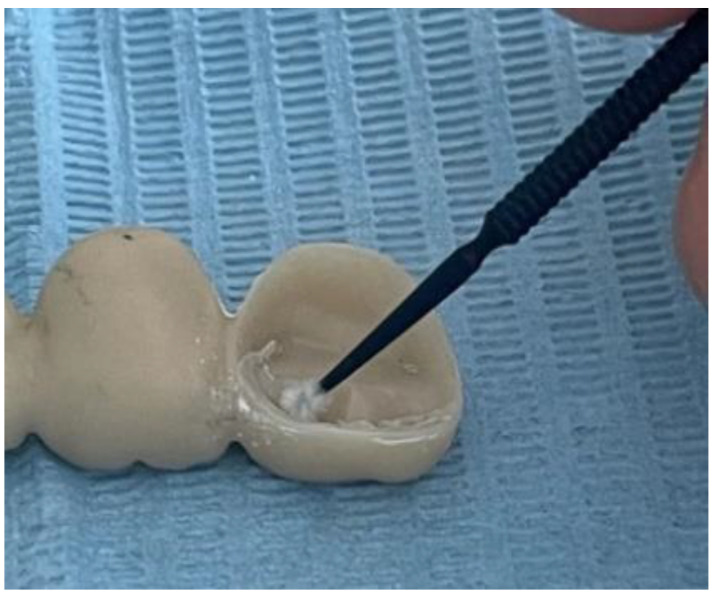
Zirconia conditioning.

Before the actual cementation, both the metal abutments and the zirconia restorations were rigorously washed and dried. Afterwards, they were degreased using ethyl alcohol to make sure no microparticles or biological residue was interfering with the cementation process. The conditioning of the zirconia restorations also consisted of applying a thin layer of Monobond Plus by Ivoclar (Figure 7). Monobond N contains an innovative combination of functional monomers that promote a strong bond to zirconium oxide, glass-ceramics, and metal alloys—comparable to the performance of bonding agents for single materials.

**Figure 8:**
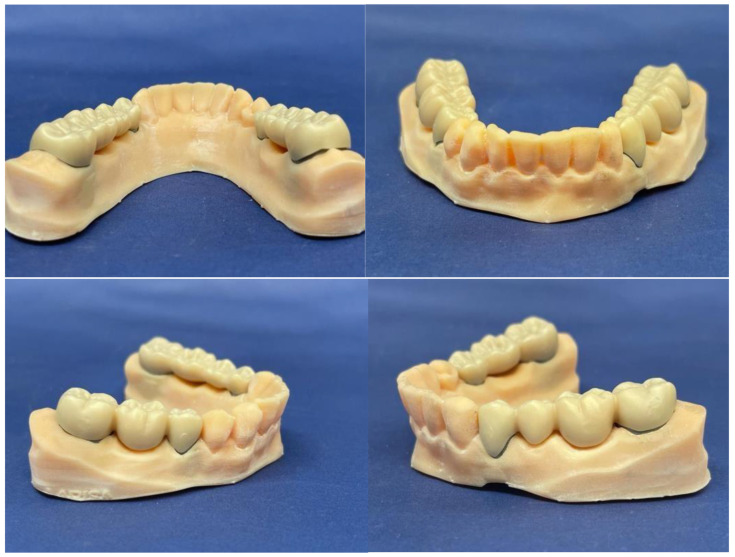
Overview of the arch model after cementing the 2 prosthetic works.

Once all the components were cleaned and conditioned, the prosthetic bridge pieces were cemented on the removable abutments (Figure 8).

The procedure entails the application of Voco’s Meron Plus QM dual cement to the inside of the dental restoration, ensuring that it is positioned accurately and securely on the surface of the restoration. Once the restoration is correctly placed, a brief light-curing process is conducted to initiate the setting of the cement. Following this initial curing, any excess cement is carefully removed using metal probes or applicators to achieve a clean finish. To complete the process, a thorough light-curing of 30 s is performed on each side of the restoration, which effectively secures it in place, ensuring optimal adhesion and stability.

For the three-unit FDPs, the connectors had dimensions of 12 mm^2^, while for the four-unit FDPs, the connector dimensions were 17 mm^2^, in accordance with manufacturer guidelines and to ensure comparability between groups.

### 2.2. Fracture Resistance Test

**Figure 9:**
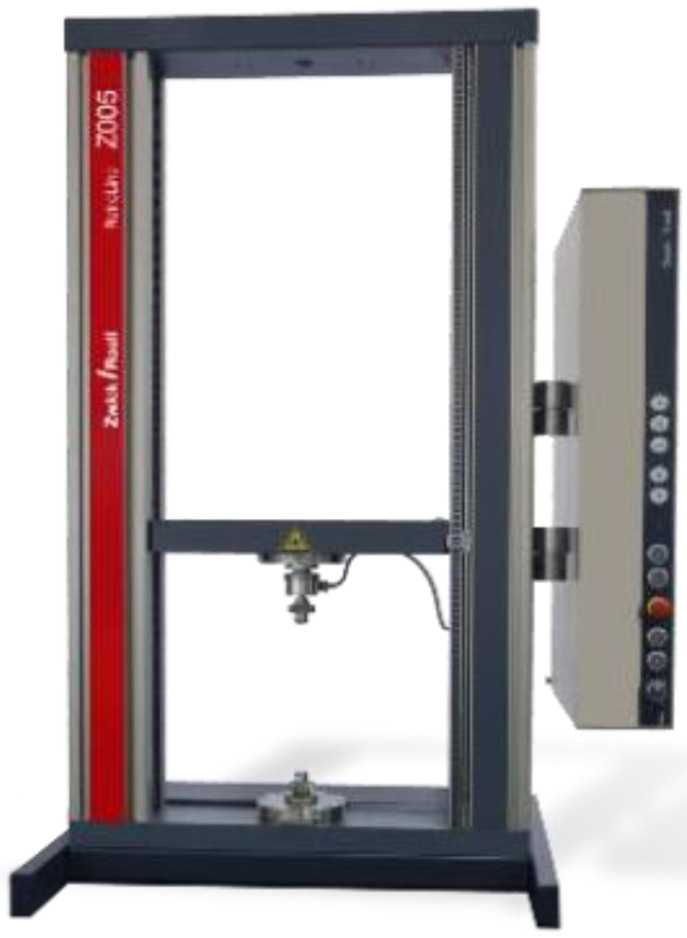
ZwichRoell ProLine Z005.

**Figure 10:**
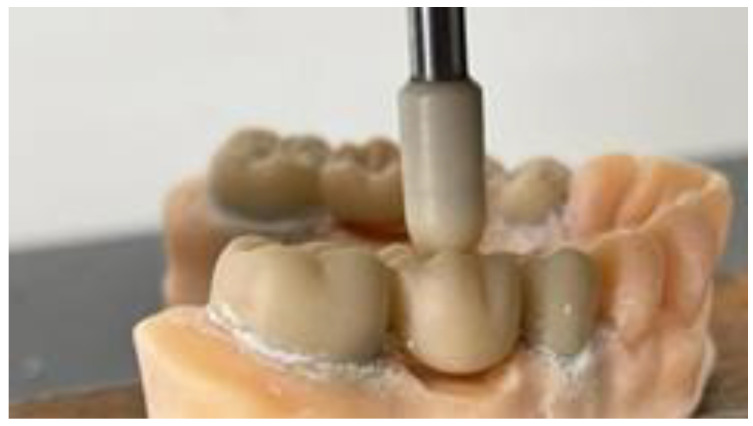
Applying occlusal force.

Following the process of cementation and allowing the cement to properly set, strength tests were conducted within the Department of Strength of Materials at the Faculty of Mechanics, Polytechnic University of Timisoara. All models were securely positioned in the ZwickRoell ProLine Z005 (ZwickRoell GmbH & Co. KG, Ulm, Germany) testing machine to apply a controlled force at a rate of 1 mm per minute through a compression test. This approach aimed to accurately determine the fracture point of each prosthetic work. The force was applied by pressing a custom-made ceramic extension directly onto the center of the occlusal face of the pontic, while the data were meticulously recorded using the ZwickRoell ProLine Z005 equipment (Figure 9 and Figure 10).

**Figure 11:**
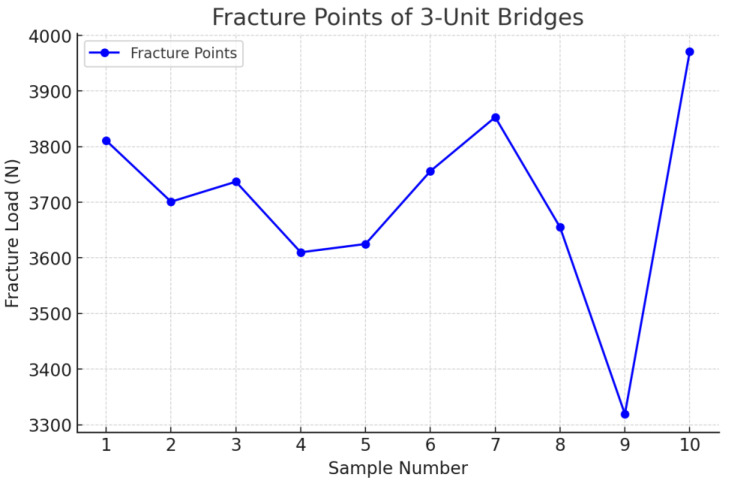
Fracture point recording the 3-unit restoration.

**Figure 12:**
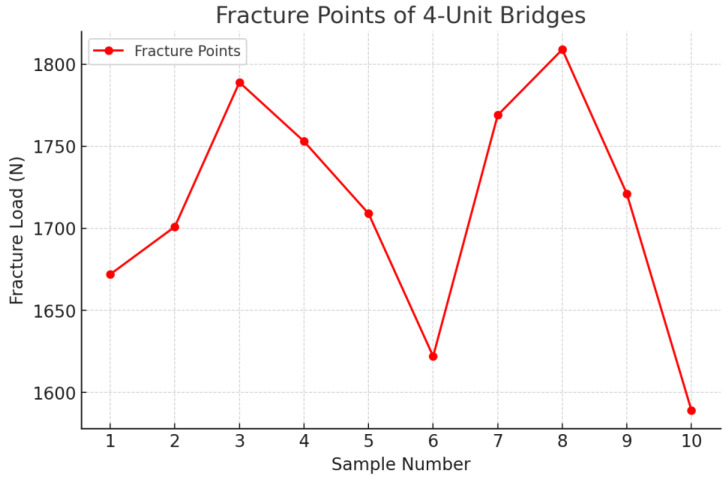
Fracture point recording the 4-unit restoration.

**Figure 13:**
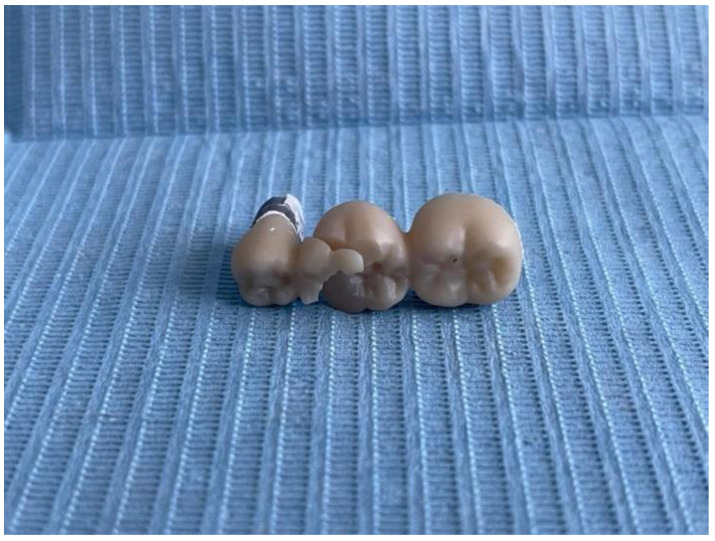
Fracture of the 3-unit restoration.

The tests were carried out for all the samples and all the fracture points values (Figure 11, Figure 12 and Figure 13) of each zirconia framework were recorded in a chart. After all the values were obtained, the statistical analysis was performed. The removable abutments used in this study were fabricated from metal alloys to ensure high durability and repeatability in mechanical testing. While natural teeth exhibit a degree of viscoelasticity and periodontal ligament damping, metal abutments provide a rigid and standardized support structure, eliminating variability due to biological differences. Although this differs from the clinical scenario, it ensures consistent mechanical loading conditions, making it suitable for comparative evaluation of fracture resistance in zirconia FDPs [18,19].

Loading Force Application and Contact Area: The occlusal force was applied at the center of the pontic to simulate maximum stress concentration in the most vulnerable area of the restoration [20]. The loading tip had a contact area of 5 mm^2^, corresponding to an anatomically relevant occlusal contact zone. The mechanical testing was performed until catastrophic failure, defined as a complete fracture of the zirconia restoration, rather than first crack detection. This approach ensures clinically relevant results, as minor cracks may not immediately compromise the restoration’s functionality, but ultimate failure does [21].

For both the three-unit and four-unit zirconia FDPs, the occlusal load was applied vertically at the center of the most distal pontic, simulating a worst-case loading scenario in the posterior region. This position was chosen to create a standardized and clinically relevant force application point, where bending stress is typically greatest. A custom-made ceramic tip with a contact area of 5 mm^2^ was used to apply the force until catastrophic failure occurred.

### 2.3. Statistical Analysis

Calculations and statistical analyses were performed utilizing MedCalc 23.0.6 for Windows (© 2024 MedCalc Software Ltd., Ostend, Belgium). To ensure the reliability of the data, tests for normal distribution were conducted using both the Kolmogorov–Smirnov test and the Shapiro–Wilk test. The means and standard deviations of the data were computed and thoroughly examined through a 2-sample t-test, alongside Levene’s Test for assessing the equality of variances. This approach allowed for a comprehensive evaluation of the mean distribution across different groups, ensuring robust statistical interpretation.

The primary variable used in the sample size calculation was the failure load (N). In a previous study [24], the failure load ranged from 936 N to 1974 N and the standard deviation was 196.37 N for the 3-unit bridges. Our preliminary data indicated a mean difference of about 170 N between the failure loads of samples of 3-unit bridges and those of 4-unit bridges. Therefore, in the sample size calculation, conducted at a statistical power of 80% and an alpha of 5%, we assumed a mean difference of 170 N and a standard deviation of the differences of 120 N. The analysis indicated a minimum requirement of 9 specimens per group; this was rounded up to *n* = 10 to ensure statistical robustness and account for potential experimental variability.

## 3. Results

At visual and microscopic inspection of the fractured samples, it was observed that the fracture typically initiated at the gingival area of the connectors, particularly in the region connecting the pontic to the abutment. In three-unit bridges, fractures were most frequently observed at the connector between the pontic and the distal abutment. In four-unit bridges, cracks consistently originated at the middle connector, between the two pontics, which was the region subjected to the highest bending stress during load application. These observations were consistent across most specimens and correlated with the stress concentration zones identified in previous studies.

Following the completion of the fracture strength tests, an analysis of the fracture zones of the zirconia restorations was conducted, along with the measurement of the specific force in newtons (N) at which each restoration fractured. For the three-unit bridge, the mean value recorded was 3703 N, while for the four-unit bridge, it was 1713 N (Table 1 and Table 2).

**Figure 14:**
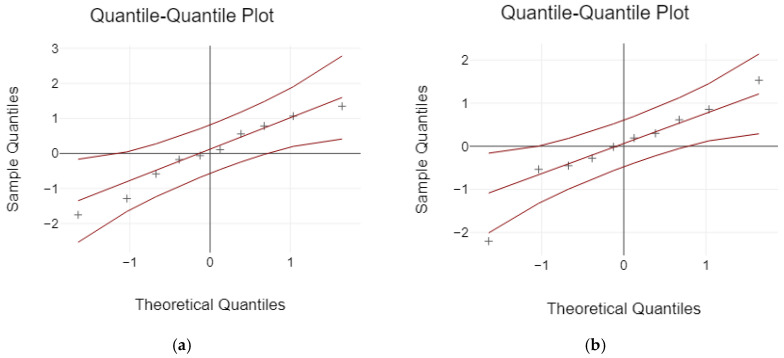
The normal distribution: (**a**) 3-unit bridges; (**b**) 4-unit bridges.

The Kolmogorov–Smirnov and Shapiro–Wilk tests were used to analyze the normal distribution of data before comparing the failure load. Based on the results, the data were not normally distributed for the failure load values (*p* < 0.05) (Table 3 and Figure 14).

The comparation statistics showed that the *3*-*unit bridges* group had higher values for the dependent variable (*M* = 3703.8, *SD* = 174.62) than the *4*-*unit bridges* group (*M* = 1713.4, *SD* = 70.94). A two-tailed *t*-test for independent samples showed that the difference between *3*-*unit bridges* and *4*-*unit bridges* with respect to the dependent variable was statistically significant, *t*(18) = 33.39, *p* = < 0.001, 95% confidence interval [1865.17, 2115.63], in the favor of the *3*-*unit bridges* (Table 4).

## 4. Discussion

The results of this study demonstrate a clear and significant influence of pontic span length on the fracture resistance of zirconia dental bridges. The shorter-span three-unit bridges showed substantially higher fracture strength (mean ~3704 N) compared to the longer-span four-unit bridges (mean ~1713 N), indicating that increasing the span length (and adding a second pontic) correlates with reduced mechanical performance. This finding is in line with previous research suggesting that longer spans in fixed prosthetics can lead to greater stress concentrations and higher likelihood of framework fracture or failure [19,20,21].

Several factors could explain the observed decrease in fracture resistance with longer pontic spans. A shorter span allows a more favorable distribution of occlusal forces across the framework. In the three-unit bridges, occlusal loads are transmitted through a single pontic to two abutments, creating a relatively rigid system where the connectors are short and robust. In contrast, the four-unit bridges have an extended section with two pontics and three connectors, which introduces more flexibility and potential bending under load. The longer span acts as a lever arm that magnifies bending moments in the framework, particularly at the connectors. As a result, the four-unit bridges likely experienced higher tensile stresses at the gingival side of the connectors during loading, leading to earlier fracture. This is consistent with our fractographic observations, where cracks often initiated at the connectors of the longer bridges. Thus, the higher fracture strength of the three-unit bridges can be attributed to their inherently greater rigidity and more efficient load transfer over a shorter distance, minimizing stress within the zirconia structure [22,23,24].

Proper framework design and connector dimensions are known to be critical for the success of zirconia fixed dental prostheses. The significant difference in performance between the two bridge designs in our study underscores that even within the manufacturer’s recommended indications (zirconia restorations with two pontics between abutment teeth) one must be cautious [25,26]. Our four-unit design was within the typical recommendation for span length, but the data clearly show a compromise in structural integrity as a result of the longer span. This aligns with the work of Lee et al. 2013 [26], who found that increasing span length (especially beyond three units) can adversely affect the fit and possibly the strength of zirconia frameworks [15]. It also resonates with other studies that have examined connector size and geometry: inadequate connector thickness or cross-sectional area can significantly weaken a bridge, particularly as spans increase [27,28,29]. In designing zirconia bridges, one should ensure that connector dimensions are appropriately scaled up when the span is increased, to provide sufficient support and reduce flexure. Our study supports this by demonstrating what can happen when a span is lengthened without a proportional change in design—the structure becomes more prone to failure [28,30,31].

The meticulous cementation and preparation protocols used in this study may have contributed to the relatively high absolute values of fracture loads observed (notably, even the weaker four-unit bridges withstood on average >1700 N, which is far above typical human bite forces). Using a quality resin-reinforced glass ionomer cement and priming the zirconia with a coupling agent likely improved the retention and support of the bridges on their abutments [14,25]. A stable, well-bonded interface means that when load is applied, the stress is distributed more evenly and the restoration behaves more like a monolithic structure together with the abutments, rather than experiencing point loads or slipping that could precipitate failure. If the bonding was insufficient, micro-movements could occur that drastically reduce the fracture resistance [26,27]. In our tests, the absence of any de-bonding or abutment-related failures suggests that the differences in fracture load are indeed attributable to the bridge design itself, not to differences in how they were retained.

From a clinical perspective, although zirconia is indicated for bridges up to five to six units, clinicians often push the envelope for longer spans due to the material’s high strength and patient demand for metal-free solutions. The present findings serve as an important reminder that even zirconia has limitations. A four-unit posterior bridge (especially involving two large pontics) may be at a higher risk of fracture in function, particularly in patients with parafunctional habits or high bite forces. It is encouraging that all our measured fracture loads (for both designs) exceed the typical maximum human bite force (which is on the order of 600–800 N in the molar region for dentate adults) [24]. This suggests that under ideal conditions, both designs might survive typical occlusal forces. However, the factor of safety is much lower for the longer-span bridges. Over time, cyclic loading (fatigue), stress corrosion, and accidental overloads could cumulatively cause a long-span zirconia bridge to fail. Indeed, the concept of mechanical fatigue is critical—while our study was a static load test to failure, in the oral environment, bridges are subject to repeated subcritical loads. Fatigue behavior can differ from static strength; zirconia, like other ceramics, can undergo slow crack growth under cyclic loads. A structure that is closer to its strength limit (as the four-unit bridges are relative to biting forces) will have a shorter fatigue life than one with ample safety margin [32,33,34,35,36].

Another important factor to consider in relation to pontic length is the marginal and internal fit of zirconia FDPs. Studies have shown that as the span length of zirconia frameworks increases (e.g., in six-unit or more extended FDPs), the precision of the marginal and internal fit may decrease [15]. This is primarily due to higher flexural deformation and potential limitations in CAD/CAM milling accuracy when fabricating longer-span zirconia restorations [16]. Poor marginal adaptation can lead to cementation gaps, plaque accumulation, and long-term prosthetic failure [17]. Therefore, understanding the impact of pontic length on both fracture resistance and adaptation accuracy is essential for optimizing zirconia FDP longevity and clinical success.

Another consideration is the role of occlusal loading patterns and patient factors. Our in vitro setup applied a centered vertical load, but clinically, off-axis loads or unilateral chewing can create uneven stress distributions. Parafunctional habits such as bruxism could impose significantly higher forces or more damaging force trajectories on a long-span bridge [37]. In such patients, the risk of fracture would be even higher for a four-unit zirconia bridge. Clinicians should evaluate patients’ occlusal schemes and consider protective measures (e.g., night guards) when long-span all-ceramic bridges are provided to patients who exhibit bruxism or other high-force conditions.

It is also worth discussing the effect of using metal abutment analogs in our tests. While metal abutments provide a consistent and stiff foundation (eliminating variability that might come from using extracted teeth or other materials), they are stiffer than natural teeth or typical implant materials [38,39]. The use of very rigid abutments might actually overestimate fracture loads because there is minimal absorption or dispersion of force by abutment deformation. In a real clinical situation, the supporting structures (tooth dentin, periodontal ligament, or even bone in case of implants) have some resilience that could lead to slightly different stress distributions. However, previous research using finite element analysis and comparative tests has shown that differences in abutment material (within a reasonable range of stiffness) do not drastically change the fracture behavior of zirconia restorations in critical areas [40,41,42]. Our choice of metal abutments likely provided an upper-bound scenario for bridge strength. It ensured that all the give was in the zirconia, not in the support. Therefore, if anything, clinical fracture loads might be a bit lower than those recorded here if there is any flex in the abutment or tooth. Nonetheless, the relative difference between a three-unit and a four-unit span should remain, as that is an intrinsic material/design property.

Overall, our findings reinforce that longer-span zirconia bridges (those exceeding three units) should be approached with caution. If a clinical scenario demands a four-unit span, modifications such as increasing connector size, using framework modifications (like strategically placed metal reinforcement or fiber reinforcement, though that somewhat defeats the metal-free intent), or splitting the span into two separate bridges (with an additional implant or abutment if possible) should be considered to mitigate the risk of failure. Additionally, ensuring an optimal occlusal scheme (minimizing heavy contacts on pontics, providing balanced contacts, etc.) can help reduce peak stresses on the prosthesis.

One limitation of this study is the inherent difference between in vitro testing and the intraoral environment. In vitro, we applied forces much higher than normal chewing forces, but we did so in a monotonic manner until failure. In the mouth, lower-magnitude forces repeated tens of thousands of times may induce fatigue. Moreover, factors like thermal cycling, the presence of saliva (which could influence slow crack growth in ceramics), and complex multidirectional forces were not reproduced. Therefore, while our static load-to-failure provides a comparative insight, it does not fully predict clinical longevity. Another limitation is that we did not include a dynamic fatigue testing phase, which would have been useful to see how many cycles each design might endure at a given load fraction of its strength. Future studies could address this by performing cyclic loading (chewing simulation) before final load to failure, which would yield more clinically relevant data on long-term performance.

While this in vitro study provides valuable insights into the relationship between pontic length and the fracture resistance of zirconia FDPs, several limitations must be acknowledged. First, the use of metal abutments and rigid resin models does not fully replicate the biomechanical behavior of natural teeth and surrounding periodontal tissues, particularly the shock-absorbing effect of the periodontal ligament. Additionally, the uniaxial loading under static conditions may not accurately reflect the complex multidirectional forces and fatigue loading patterns encountered in the oral environment over time. Another limitation is the sample size, which, while sufficient for detecting significant differences in fracture resistance, may not fully capture the variability present in clinical scenarios. Given these constraints, future studies should involve dynamic fatigue testing, thermocycling, and finite element analysis to better simulate intraoral conditions. Moreover, clinical trials and longitudinal cohort studies are essential to validate these findings in real-world settings and to investigate how pontic length interacts with occlusal scheme, parafunctional habits, and bone support. Despite these limitations, the present results hold strong clinical relevance: they underscore the need for caution when designing extended-span zirconia FDPs, as increased pontic length significantly compromises structural integrity. By recognizing these biomechanical limitations, clinicians can make more informed decisions regarding case selection, material choice, and connector dimensioning, ultimately improving the longevity and success of zirconia prosthetic treatments.

Despite these limitations, the stark contrast observed between the two designs provides valuable guidance. It underscores a mechanical principle: as the span length increases, the bending forces escalate, thereby lowering the fracture threshold of brittle materials like zirconia.

## 5. Conclusions

Within the limitations of this in vitro study, the following key findings were observed:

- Pontic length has a direct and significant impact on the fracture resistance of zirconia FDPs. Three-unit zirconia FDPs with a pontic span of 11 mm exhibited a mean fracture resistance of 3703 N, significantly higher than four-unit FDPs.
- Four-unit zirconia FDPs, with a pontic span of 17 mm, demonstrated a mean fracture resistance of 1713 N, indicating reduced structural integrity with increased span.
- All tested values exceeded the average human bite force, but the risk of long-term mechanical fatigue increases with extended spans.
- The findings support limiting pontic length in zirconia bridges to optimize durability and minimize the risk of mechanical failure.
- These results are clinically relevant for guiding prosthodontic planning, particularly in cases involving posterior multi-unit zirconia restorations.

## Figures and Tables

**Table 1:** Fracture points of the 3-unit bridges.

Bridge	Fracture Point
1	3811
2	3701
3	3737
4	3610
5	3625
6	3756
7	3853
8	3655
9	3319
10	3971
Mean value	3703.8

**Table 2:** Fracture points of the 4-unit bridges.

Bridge	Fracture Point
11	1672
12	1701
13	1789
14	1753
15	1709
16	1622
17	1769
18	1809
19	1721
20	1589
Mean value	1713.4

**Table 3:** Test for normality for the load-to-failure (N) values.

	Statistics	*p* Value
Kolmogorov–Smirnov	0.2	0.771
Shapiro–Wilk	0.94	0.545

**Table 4:** Descriptive statistics of the load to failure (N) according to the number of -units.

	*n*	Mean	SD	Minimum	Maximum	*p* Value
3-unit bridges	10	3703.8	174.62	3319	3971	<0.001 ^a^
4-unit bridges	10	1713.4	174.62	1589	1809	

^a^ *t*-test.

## Data Availability

The data presented in this study are available on request from the corresponding author.

## Abbreviations

The following abbreviations are used in this manuscript:

N	Newton
CAD	computer-aided design
CAM	computer-aided manufacturing
MPa	megapascal
3D	three-dimensional
SD	standard deviation
n	number
